# Multi-Layer Low Earth Orbit Constellation Capacity Fundamental

**DOI:** 10.3390/s26134059

**Published:** 2026-06-26

**Authors:** Shaofan Hu, Min Sheng, Di Zhou, Jiandong Li

**Affiliations:** State Key Laboratory of Integrated Service Networks, Institute of Information Science, Xidian University, Xi’an 710071, China

**Keywords:** capacity analysis, multi-layer low Earth orbit constellation, bottleneck link

## Abstract

Multi-layer low Earth orbit constellations (ML-LEOs) have become a pivotal trend in the development of satellite network systems, where their layered orbital architecture improves system performance by strategically deploying satellites in distinct orbital layers. However, two critical issues remain open: how does the configuration of ML-LEO affect its performance, and how many layers are required to achieve optimal performance? This paper first investigates the impact of the number of layers *L* on the capacity of ML-LEOs. By analyzing the distribution of inter-layer inter-satellite links (ISLs) and the flow count on bottleneck links, we derive a closed-form mathematical expression for ML-LEO capacity under different values of *L*. In particular, we show that when each layer adopts an identical constellation topology and the number of satellites per orbit equals the number of orbits, the capacity of the ML-LEO is $\sqrt{L}$ times that of a single-layer low Earth orbit constellation (SL-LEO). Furthermore, we present the optimal parameter configuration for ML-LEOs: the number of orbits per layer should equal the number of satellites per orbit, the number of layers should be half the number of satellites per orbit, and the optimal number of inter-layer ISLs is twice the product of the number of orbits per layer and the number of layers. Finally, extensive simulations are carried out to thoroughly verify the accuracy of the analytical results. Our analysis reveals the performance benefits of multi-layer topology and establishes a theoretical framework for parameter optimization in ML-LEO.

## 1. Introduction

**Figure 1:**
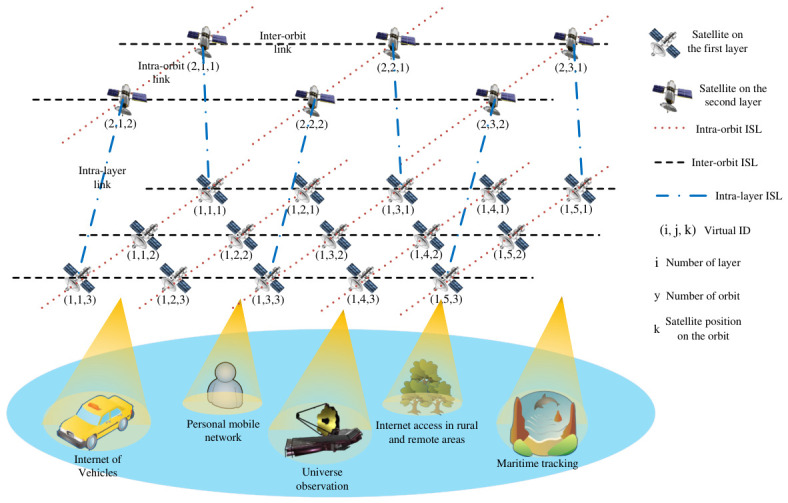
Now and future application scenarios for ML-LEOs.

Multi-layer low Earth orbit constellations (ML-LEOs) organize satellites at the same orbital altitude into a layer to address today’s wide variety of communications services, such as Internet access in rural and remote areas, Internet of Vehicles, disaster warning and rescue, e-commerce and astronomical observation [1]. In addition, with the surge in global data, future technologies such as the Internet of Space Things, low-power long-range networks, universe observation, aeronautical communication and maritime tracking will also rely more on ML-LEOs for service provision [2]. Therefore, systems such as the Telesat system, Kuiper system and Starlink system have adopted the multi-layer topology [3]. Figure 1 shows some application scenarios for ML-LEOs [4].

Many related studies have focused on the configuration design of satellite constellations based on various performance metrics. The fundamental mathematics enabling the constellation design of the Flower Constellation is provided in [5]. The study in [6] derives a coverage probability model for multi-layer multi-altitude LEO constellations via stochastic geometry, compensates for non-homogeneous satellite distribution and provides VLEO parameter insights. In addition, the study in [7] proposes a dense LEO constellation configuration optimization method tailored for inter-satellite interference mitigation, which optimizes orbital arrangement and satellite distribution to suppress co-channel interference while guaranteeing network coverage and communication performance. The study in [8] presents a QoS-driven satellite constellation design strategy for LEO satellite Internet of Things, which optimizes orbital configuration and satellite layout to simultaneously satisfy coverage, communication quality of service, and system deployment cost constraints.

The capacity of ML-LEOs is a critical performance metric for evaluating the system’s efficiency [9]. Investigating the capacity of ML-LEOs helps optimize constellation performance, ensuring their capability to support present and future data traffic demands. From the perspective of network planning and design, network capacity is divided into access capacity and throughput capacity. Throughput capacity refers to the total amount of data that the network infrastructure, such as satellites and inter-satellite links (ISLs) in ML-LEOs, can process and transmit within a given time, typically measured in Gbps or Tbps. Throughput capacity is influenced by factors including the bandwidth of network links, the processing capabilities of network devices, and the network topology. This metric characterizes the overall transmission efficiency, signal processing capability, and topological performance of the satellite network. Unlike throughput capacity, which pertains to the overall capabilities of the network infrastructure, access capacity is specific to each individual connection or access point within the network. It is defined as the capability of individual users, devices, or premises to connect to and utilize network resources, typically measured in terms of bandwidth. This metric reflects the performance of the satellite network when serving a single user and is influenced by the bandwidth of the user link, the configuration of the ground station, and the reception capability of the user terminal.

Regarding access capacity, studies [10,11] mainly analyzed the impacts of forward links and terrestrial networks in SL-LEO systems, respectively. The access capacity analysis of ML-LEOs has also been investigated in [12], which employs both stochastic geometry and queueing theory to minimize the total number of satellites. The study in [13] explored the relationship between coverage rate, multiple coverage, and average coverage over the target area. In addition, most capacity-oriented constellation optimization algorithms also focus on access capacity. These methods include a distributed resource allocation mechanism based on the alternating direction method of multipliers, a genetic algorithm for gateway placement, and a capacity-oriented constellation design method with multiple optimization objectives [14,15,16,17]. Evidently, algorithms for access capacity analysis are tailored to specific components of constellations, such as the performance of uplinks and downlinks. However, evaluating throughput capacity requires a holistic approach that takes the entire constellation into account, including key factors such as constellation topology, available bandwidth, and traffic models. Therefore, algorithms designed for access capacity analysis are not applicable to the evaluation of throughput capacity.

Throughput capacity is directly related to user experience, thus playing a pivotal role in network planning, optimization, and management [9]. In addition, the improvement of throughput capacity for the entire satellite network relies heavily on large-scale infrastructure upgrading and system-level collaborative optimization, which entails greater technical complexity and poses practical implementation challenges. A network may have high access capacity, but if the throughput capacity of the entire network is insufficient to support the demands of all users, the network will still suffer from congestion and performance degradation. By evaluating throughput capacity, administrators can determine the necessity of equipment upgrades, network topology adjustments, or bandwidth expansion to preemptively alleviate congestion and avoid performance degradation. The study of throughput capacity is essential for ensuring the continuous operation of the network and enhancing user experience. ML-LEOs offer significant advantages in improving throughput capacity. First, the multi-layer topology of ML-LEOs enables spatial reuse and hierarchical coverage, allowing for denser satellite deployment without increased intra-layer interference, thereby improving throughput capacity. In addition, the multi-layer topology enables more diverse ISLs and more advanced routing strategies, which reduce the number of transmission hops and further enhance throughput capacity. To provide a theoretical basis for the throughput capacity of ML-LEOs, it is of great intrinsic interest to explore how the constellation configuration should be designed to achieve superior capacity performance. This issue motivates us to further investigate the following subquestions:

- Compared to SL-LEOs, ML-LEOs increase the number of available ISLs, thereby leveraging the spatial dimension more effectively. Such optimization of the constellation topology helps improve throughput capacity. Nevertheless, a critical question arises: given a fixed total number of satellites, does a positive correlation consistently exist between throughput capacity and the number of layers? Compared to SL-LEOs, ML-LEOs increase the number of available ISLs, thereby enabling more effective spatial reuse. This enhanced connectivity helps improve throughput capacity. However, a critical question remains: when the total number of satellites is fixed, does throughput capacity always increase with the number of layers?
- The intra-layer topology of each layer in an ML-LEO, typically modeled as a 2D-torus network, is characterized by the number of orbits and satellites per orbit. These parameters govern intra-layer connectivity and routing efficiency, thereby significantly influencing overall constellation capacity. Therefore, the second subquestion is: with a fixed total number of satellites and a fixed number of layers, what design principles should be followed to optimize the intra-layer topology for maximum capacity?
- Once the number of layers and their intra-layer topologies in an ML-LEO are determined, another critical factor is the number of inter-layer ISLs. Although more inter-layer ISLs can reduce data transmission hops and thereby improve capacity, the goal is to achieve the target throughput with as few inter-layer ISLs as possible. Accordingly, the third question is, how should a reasonable inter-layer ISL configuration be designed?

Existing capacity analyses mainly focus on SL-LEOs, with limited investigations into ML-LEOs. Furthermore, none of the prior multi-layer studies derived closed-form capacity expressions that directly reveal the relationship between ML-LEO parameters and throughput capacity. The study in [18] analyzes the throughput capacity of SL-LEOs under the all-to-all traffic model based on the maximum flow minimum cut (MFMC) algorithm. However, the MFMC algorithm cannot regard all satellites as both sources and destinations since it aggregates all source satellites into a single super source and all destination satellites into a single super destination. In this case, all satellites are treated as a single super node, leading to an infinite calculated throughput capacity, which is unrealistic. Since the topology of SL-LEOs is a 2D torus, the analysis in [18] is valid for the 2D-torus topology of SL-LEOs. The study in [19] also focuses on SL-LEOs and proposes a throughput capacity maximum topology design algorithm to determine constellation parameters and connection relationships, which minimizes the average path length of data packet transmission and maximizes the utilization rate of each ISL, thereby achieving the upper bound of throughput capacity. In addition, the study in [20] establishes an analytical capacity model for LEO mega-constellations with quasi-torus topologies, derives closed-form capacity expressions under inter-satellite link pruning, and reveals that quasi-torus topology with fewer ISLs can maintain nearly optimal network capacity while reducing system deployment and hardware costs. The study in [21] analytically derives the exact outage probability and its approximated expression using the Poisson limit theorem. Based on these derived expressions, the system throughput maximization problem is formulated, and an iterative algorithm is proposed to obtain near-optimal solutions under constraints of satellite visibility and outage probability. The throughput capacity of two-layer constellations is investigated in [22]. This study only considers all satellites on the lower layer as both sources and destinations, while satellites on the upper layer act merely as relays rather than sources or destinations.

This paper mainly investigates the throughput capacity gain brought by ML-LEOs, focusing on the relationship between throughput capacity and key parameters of ML-LEOs, including the number of layers, the number of orbits and satellites per orbit on each layer, and the number of inter-layer ISLs. Based on the analysis, recommendations for the optimal parameter configuration of ML-LEOs are provided. The major contributions of this paper are summarized as follows:

- Capacity Analysis of ML-LEOs: For each layer, the relative positions of inter-layer ISLs connecting satellites on the upper and lower layers also affect capacity. To quantitatively characterize this distribution, we first define an inter-layer coupling coefficient. We then classify all flows according to their sources and destinations, which enables us to analyze the number of flows passing through bottleneck links—a quantity inversely proportional to throughput capacity. On this basis, we perform a thorough throughput capacity analysis of ML-LEOs.
- Optimal Parameter Configuration: Considering *L*-layer ML-LEOs with identical constellation topologies across layers, where the number of satellites per orbit equals the number of orbits, we prove that the throughput capacity of ML-LEOs is $\sqrt{L}$ times that of an SL-LEO with the same total number of satellites. Furthermore, throughput capacity is maximized when *L* equals half the number of orbits. In addition, we analyze the impacts of the number of inter-layer ISLs and ISL data rate on throughput capacity, and propose a balanced configuration of these two factors to achieve optimal performance.
- Simulation Verification: We conduct extensive numerical simulations for typical ML-LEO scenarios to verify the accuracy of the proposed analytical framework. The results illustrate the influence of key parameters on ML-LEO capacity, thereby validating the effectiveness of our method. All these analyses provide valuable insights for the performance evaluation and system design of ML-LEOs.

The rest of this paper is organized as follows. In Section 2, we establish the system model for ML-LEOs, including the satellite coverage model, the intra-layer topology model, and the inter-layer topology model. In addition, we introduce the traffic model and the routing strategy. Section 3 presents a detailed throughput capacity analysis of ML-LEOs. In Section 4, we investigate the capacity gain and derive the optimal parameter configuration for ML-LEOs. To verify the validity of our analysis, comprehensive numerical simulations are conducted in Section 5. Finally, Section 6 concludes this paper. The main notations used throughout this paper are summarized in Table 1.

## 2. System Model

**Figure 2:**
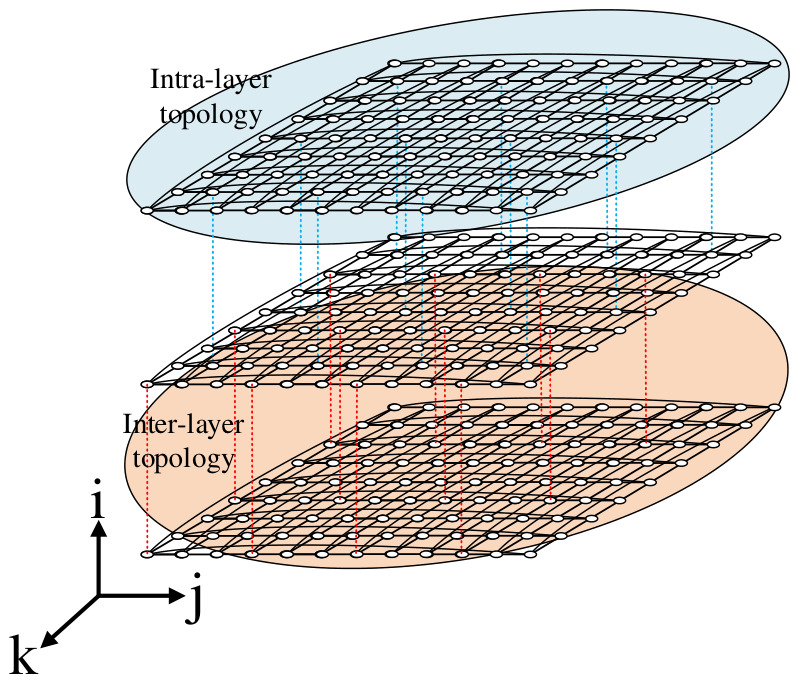
A topology of ML-LEO with $N_{1}=N_{2}=N_{3}=12$ and $M_{1}=M_{2}=M_{3}=9$.

Consider an ML-LEO constellation with *T* satellites and *L* layers in total. Let $N_{i}$ be the number of orbits on the *i*-th layer, and $M_{i}$ the number of satellites per orbit on the *i*-th layer. Satellites are uniformly distributed along their respective orbits across all layers and interconnected via ISLs as shown in Figure 1 and Figure 2. An ISL connecting satellites on the same orbit is referred to as an intra-orbit ISL, while an ISL between satellites on different orbits is called an inter-orbit ISL. For brevity, intra-orbit and inter-orbit ISLs are collectively termed intra-layer ISLs. In addition, an ISL that links satellites belonging to different layers is defined as an inter-layer ISL. Accordingly, the topology formed by satellites in the same layer connected via intra-layer ISLs is called the intra-layer topology, and the topology formed by satellites on adjacent layers linked through inter-layer ISLs is defined as the inter-layer topology. In this section, we first present the satellite coverage model, as it serves as the foundation for establishing inter-layer ISLs between satellites on adjacent layers. We then introduce the classic intra-layer topology model and its convenient representation to facilitate the following analysis. The inter-layer topology model is also presented to form the complete ML-LEO topology. Finally, we propose the adopted traffic model and routing strategy based on the characteristics of ML-LEO constellations.

### 2.1. Satellite Coverage Model

**Figure 3:**
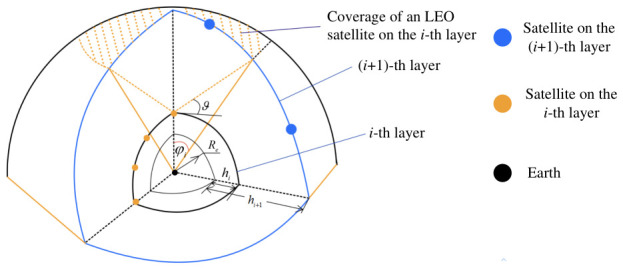
Inter-layer ISL model.

The necessary prerequisite for establishing ISLs is unobstructed line-of-sight between the communicating satellites, and the target satellite lies within the coverage of the transmitting satellite. To ensure that satellites on the *i*-th layer can establish connections with their nearest neighbors on the $\left(i+1\right)$-th layer, we require that the coverage of satellites on the *i*-th layer covers the nearest satellites on the $\left(i+1\right)$th layer. The coverage of satellites on the *i*-th layer is illustrated in Figure 3 [22]. The coverage range of a satellite on the *i*-th layer can be characterized by its half-side center angle $\varphi_{i}$. $\varphi_{i}$ is defined as the angular distance from the satellite on the *i*-th layer to the edge of its coverage, which is designed to cover the nearest satellite on the $\left(i+1\right)$-th layer. The expression for $\varphi_{i}$ is given in Equation (1):

(1) $$\varphi_{i}=\frac{\pi }{2}-\vartheta_{i}-\arcsin\left(\frac{h_{i}+R_{e}}{h_{i+1}+R_{e}}\cos\vartheta \right),$$

where $h_{i}$ is the altitude of satellites on *i*-th layer, $h_{i+1}$ represents the altitude of satellites on the $\left(i+1\right)$-th layer and $\vartheta_{i}$ denotes the minimum elevation angle of the antenna for the *i*-th layer satellites. In addition, $R_{e}$ represents the radius of the earth.

To satisfy the requirements of the inter-layer topology, we derive the following constraint on satellite parameters via geometric derivation:

(2) $$\varphi_{i}>\arccos\left(\cos\frac{\omega_{Y_{i+1}}}{2}\cos\Theta_{max}\left(i\right)\right),$$

where $\Theta_{i}\left(j\right)$ denotes the angle between the *j*-th LEO orbital plane on the *i*-th layer and the orbital plane on the $\left(i+1\right)$-th layer with which ISLs are established, where $1\leq j\leq N_{i}$. In addition, $\omega_{Y_{i}}=2\pi /M_{i}$ is the angular distance between adjacent orbits on the *i*-th layer.

The geometric visibility condition (2) ensures that satellites in adjacent layers are within the line of sight. However, for laser-based inter-layer ISLs, the link establishment involves acquisition, pointing, and tracking (ATP). Let $\omega_{\mathrm{rel}}\left(i,i+1\right)$ denote the maximum relative angular velocity between a satellite on the *i*-th layer and its visible counterparts on the (i+1)-th layer. The ATP system must satisfy

(3) $$\omega_{\mathrm{rel}}\left(i,i+1\right)\leq \omega_{\max},$$

where $\omega_{\max}$ is the maximum tracking angular velocity of the laser terminal. Furthermore, let $\tau_{\mathrm{hold}}\left(i,i+1\right)$ denote the minimum link holding time, defined as the duration during which the angular separation remains within the ATP acquisition field of view $\theta_{\mathrm{FOV}}$:

(4) $$\tau_{\mathrm{hold}}\left(i,i+1\right)\geq \tau_{\mathrm{acq}}+\tau_{\min},$$

where $\tau_{\mathrm{acq}}$ is the acquisition time and $\tau_{\min}$ is the minimum stable transmission duration.

When (3) and (4) are satisfied, the inter-layer ISL operates at full data rate $B_{z}$. Otherwise, the effective capacity is degraded by an ATP efficiency factor:

(5) $$\eta_{\mathrm{ATP}}=\frac{\tau_{\mathrm{hold}}-\tau_{\mathrm{acq}}}{\tau_{\mathrm{hold}}}\cdot I\left(\omega_{\mathrm{rel}}\leq \omega_{\max}\right),$$

where I(·) is the indicator function. The ATP-constrained inter-layer ISL rate is

(6) $${\tilde{B}}_{z}=\eta_{\mathrm{ATP}}\cdot B_{z}.$$

In the subsequent capacity analysis, replacing $B_{z}$ with ${\tilde{B}}_{z}$ yields the ATP-constrained throughput capacity. For the scenarios considered in this paper (adjacent layer altitude separation Δh∈[50,100] km, circular orbits), $\omega_{\mathrm{rel}}$ is on the order of $10^{-3}$–$10^{-2}$ rad/s, well within the tracking capability of state-of-the-art laser terminals ($\omega_{\max}\approx 1$ rad/s [23]). Thus $\eta_{\mathrm{ATP}}\approx 1$ and the unconstrained capacity serves as a reasonable bound.

### 2.2. Intra-Layer Topology Model

The intra-layer topology is typically constructed as a 2D torus network in most related studies [18,22,24]. This is because satellites on each layer are uniformly distributed along their respective orbits. Treating each orbit as a circular path, the satellite distribution along orbits presents periodicity. As a satellite moves along its orbit, it returns to its initial position after one full revolution, which is analogous to the unidirectional periodicity of a 2D torus. In addition, the satellite distribution within each layer of the constellation can be regarded as an arrangement on an orbital ring. Although the actual satellite motion takes place in 3D space, the spatial distribution of satellites along orbits shows topological characteristics consistent with those of a 2D torus.

Consider the intra-layer topology of the *i*-th layer. Each *i*-th layer contains $T_{i}=N_{i}\times M_{i}$ satellites, which are interconnected via intra-layer ISLs to form an $N_{i}\times M_{i}$ 2D torus topology as shown in Figure 2. We use solid lines to represent intra-layer ISLs. Due to the mobility of satellites, we employ the virtual ID (i,j,k) shown in Figure 1 to denote satellites. Assigning each satellite a virtual ID (i,j,k) facilitates the characterization and analysis of the constellation. Here, *i* and *j* denote the layer number and the orbit number of the satellite, respectively, and *k* represents the position of the satellite on this orbit. Notably, the satellite corresponding to a specific virtual ID is not fixed. Instead, the satellite that moves into this position inherits the virtual ID. This method reduces the analytical complexity introduced by satellite mobility [22].

### 2.3. Inter-Layer Topology Model

The inter-layer topology connects the intra-layer topologies of adjacent layers. We adopt a uniformly distributed inter-layer ISL model as shown in Figure 2. The uniform distribution of inter-layer ISLs is justified by three facts. First, the coverage condition (2) ensures that each satellite has multiple visible candidates on the adjacent layer, making uniform selection feasible. Second, satellites in each layer are uniformly distributed along orbits, and orbits are uniformly distributed in space, constructing a 2D-torus topology. The periodic satellite motion yields a periodic inter-layer visibility pattern, enabling a stable uniform topology. Third, under all-to-all traffic, uniform distribution equalizes the load across all inter-layer ISLs. Since the throughput capacity is inversely proportional to the maximum flow count on any single link, load balancing maximizes capacity. Any non-uniform distribution would concentrate flows on certain links, increasing the maximum per-link load and thus reducing the throughput capacity.

In order to characterize the uniform distribution of inter-layer ISLs, we first define $n_{\left(i,i+1\right)}$ as the number of inter-layer ISLs between the *i*-th and $\left(i+1\right)$-th layers, where $n_{\left(i,i+1\right)}=a_{\left(i,i+1\right)}\times b_{\left(i,i+1\right)}$. Here, $a_{\left(i,i+1\right)}$ and $b_{\left(i,i+1\right)}$ represent the decomposition of $n_{\left(i,i+1\right)}$ across orbits and along orbits, respectively. However, the relative position between inter-layer ISLs remains to be defined to complete the model.

**Definition** **1.** *(Key Satellite): Key satellites denoted by κ are those satellites that have inter-layer ISLs.*

We denote key satellites on the i-th layer that connect to the $\left(i-1\right)$-th layer as $\kappa_{\left(i,i-1\right)}$, and those that connect to the $\left(i+1\right)$-th layer as $\kappa_{\left(i,i+1\right)}$. Based on these definitions, we describe the characteristic and corresponding analysis as follows.

**Figure 4:**
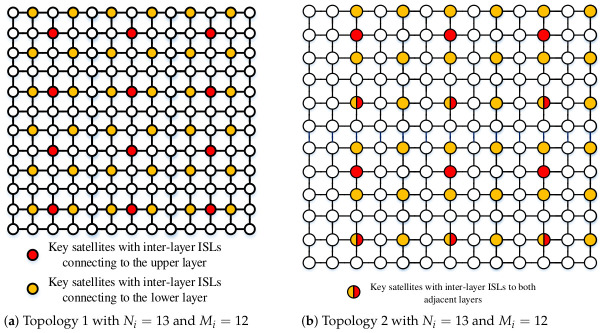
Illustration of analysis of $I\left(i\right)$ and $\alpha_{i}$.

(1) It is obvious that for two-layer ML-LEOs, in addition to the intra-layer ISLs, the satellites on the first layer will only have inter-layer ISLs connecting the satellites on the upper layer, and the satellites on the second layer will only have inter-layer ISLs connecting the satellites on the lower layer. This means that each layer has only one of $\kappa_{\left(i,i+1\right)}$ or $\kappa_{\left(i,i-1\right)}$. However, for ML-LEOs with three or more layers, the *i*-th layer (where 1<i<L) contains both $\kappa_{\left(i,i+1\right)}$ and $\kappa_{\left(i,i-1\right)}$. Furthermore, $\kappa_{\left(i,i+1\right)}$ and $\kappa_{\left(i,i-1\right)}$ may overlap, i.e., the same satellite may serve as a gateway to both adjacent layers. It is worth noting that this phenomenon not only affects the topology of ML-LEOs but also affects the transmission of flows. To illustrate, consider a key satellite on the *i*-th layer. When a satellite on the upper layer communicates with a satellite on the lower layer via the inter-layer ISLs of this key satellite, if the key satellite on the *i*-th layer has inter-layer ISLs connecting both the $\left(i+1\right)$-th layer and the $\left(i-1\right)$-th layers, these flows do not pass through the intra-layer ISLs on the *i*-th layer and thus do not consume their bandwidth. Otherwise, they will. In response to this phenomenon, we divide all ML-LEOs into:
  Case 1: ML-LEOs with L=2.
  Case 2: ML-LEOs with L>2.
(2) For the *i*-th (1<i<L) in Case 2, even when the constellation size, intra-layer topology, and inter-layer ISL count between adjacent layers are fixed, the relative position of inter-layer ISLs remains undetermined if only uniform ISL distribution is specified. Figure 4 illustrates this phenomenon. For clarity, we omit wrap-around ISLs between the first and last satellites on each orbit, as well as between corresponding satellites on the first and last orbits; the same convention applies to subsequent figures. Figure 4a,b both show the *i*-th layer with $N_{i}=13$ and $M_{i}=12$. Red and orange nodes denote $\kappa_{\left(i,i+1\right)}$ and $\kappa_{\left(i,i-1\right)}$, respectively. In Figure 4b, bicolored nodes represent satellites with inter-layer ISLs connecting both the (i−1)-th and (i+1)-th layer. Both figures contain 30 $\kappa_{\left(i,i+1\right)}$ and 12 $\kappa_{\left(i,i-1\right)}$. However, even if the above parameters are the same, the topologies of the *i*-th layer in Figure 4a,b are different: in Figure 4a the two sets are disjoint, while in Figure 4b they overlap. This difference arises because uniform ISL deployment only determines the positions of $\kappa_{\left(i,i+1\right)}$ and $\kappa_{\left(i,i-1\right)}$ independently but not their relative position. To quantify this relative position, we define the inter-layer coupling coefficient $\alpha_{\left(i,i+1\right)}$ and $\alpha_{\left(i,i-1\right)}$ for the *i*-th layer.

**Definition** **2.** *(Inter-layer Coupling Coefficient): The inter-layer coupling coefficient between the i-th and the $\left(i+1\right)$-th layer, denoted by $\alpha_{\left(i,i+1\right)}$, is the ratio of the number of satellites on the i-th layer that connect to both the $\left(i-1\right)$-th and $\left(i+1\right)$-th layers to the total number of satellites on the i-th layer that connect to the $\left(i+1\right)$-th layer, i.e., $\alpha_{\left(i,i+1\right)}=\frac{n_{i}}{n_{\left(i,i+1\right)}}$, where $n_{i}$ is the number of satellites on the i-th layer that connect to both the $\left(i+1\right)$-th and $\left(i-1\right)$-th layers simultaneously.*

### 2.4. Traffic Model and Route

Users access their nearest available satellite, and each satellite serves as a data source that transmits information to all other satellites in the constellation. The all-to-all traffic model is adopted for ML-LEOs, reflecting the full-mesh connectivity and uniform service demands of such systems [18,24,25]. For a network with *T* nodes, this model assumes that all nodes originate equal traffic volumes and function as both sources and destinations. Each source node distributes its traffic uniformly among the other T−1 nodes, transmitting one unit to each.

It should be noted that the all-to-all traffic model used here is a theoretical benchmark. It helps us understand the maximum throughput of the ML-LEO system when traffic demand is the same everywhere. This model works well when (i) the satellite network covers the whole world with the same number of users everywhere, (ii) most traffic goes between satellites rather than to the ground, such as remote sensing data relay or data center syncing, or (iii) the system is built to handle the busiest possible time. But in real life, LEO networks usually have uneven traffic. Some areas have more users than others, and satellite beams are fixed in shape, which was pointed out in [26]. In these cases, the capacity under the all-to-all traffic model can still serve as a benchmark to provide guidance.

For routing, a minimum hop routing strategy is adopted when the source and destination satellites are in the same layer [27,28]. When the source and destination are not on the same layer, since intra-layer ISLs are more stable than inter-layer ISLs, the routing strategy prioritizes transmitting flows through intra-layer ISLs from the source satellite as much as possible (subject to the minimum hop rule), and then via inter-layer ISLs in each intermediate layer to the destination satellite. For a source satellite $\left(i_{s},j_{s},k_{s}\right)$ and a destination satellite $\left(i_{d},j_{d},k_{d}\right)$, the routing path is $\left(i_{s},j_{s},k_{s}\right)$→⋯→$\left(i_{s},j_{s}^{kd},k_{s}^{kd}\right)$→⋯$\to \left(i_{s}\pm 1,j_{s+1}^{kd},k_{s+1}^{kd}\right)\to \cdots \to \left(i_{d},j_{d}^{kd},k_{d}^{kd}\right)\to \cdots \to \left(i_{d},j_{d},k_{d}\right)$, where $\left(i_{s},j_{s}^{kd},k_{s}^{kd}\right)$ denotes the key relay satellite on the $i_{s}$-th layer, which is the nearest satellite with inter-layer ISLs to the destination satellite. For subsequent capacity analysis, we call satellite $\left(i_{s},j_{s}^{kd},k_{s}^{kd}\right)$ the key relay satellite on the $i_{s}$-th layer. The notations for other intermediate satellites in the routing path follow the same rule.

## 3. Analysis of the ML-LEO Capacity

### 3.1. Fundamentals of the Capacity

Before conducting throughput capacity analysis, several definitions need to be clarified.

**Definition** **3.** *(Flow): A flow ${Ϝ}_{\left(s,d\right)}\;\left(s\neq d\right)$ is defined as a data flow traversing the network from the source node s to the destination node d.*

**Definition** **4.** *(Maximum achievable flow): The maximum achievable flow F is defined as the maximum amount of flow that can be transmitted from a source node to a destination node in a network.*

We use $\zeta_{\left(s,d\right)}$ to denote the value of ${Ϝ}_{\left(s,d\right)}$, where ${Ϝ}_{\left(s,d\right)}$ is the actual flow from source node *s* to destination node *d*. Similarly, we denote the magnitude of the maximum achievable flow F by ξ.

**Definition** **5.** *(Network Throughput Capacity): The throughput capacity is defined as the sum of the maximum value of all flows in the network [29], i.e.,*

(7) $$C=\sum_{s=1}^{T}\sum_{d=1}^{T}\max\left(\zeta_{\left(s,d\right)}\right),\;s\neq d.$$

*As for the ALL-to-ALL traffic model, the network throughput capacity can be expressed as the product of the number of flows and the maximum achievable flow as Equation (8) because $\zeta_{\left(s,d\right)}=\xi$ [22].*

(8) $$C=T\left(T-1\right)\xi$$

To facilitate further analysis, we first present additional definitions and then derive the corresponding theorems. For simplicity, the term “capacity” in subsequent discussions specifically refers to the throughput capacity.

**Definition** **6.** *(Bottleneck Link): A bottleneck link is defined as the link that carries the maximum number of flows in the network (i.e., the most heavily loaded link).*

Here, we use χ to denote the number of flows passing through a bottleneck link. Notably, a network may contain multiple bottleneck links. We use $B_{xy}$ to represent the transmission rate of intra-layer ISLs and $B_{z}$ to represent that of inter-layer ISLs. We first set $B_{xy}=B_{z}=B$. In the next section, we will analyze the case where $B_{xy}\neq B_{z}$.

**Theorem** **1.** *The network throughput capacity can be expressed in a concise and fundamental form as Equation (9):*

(9) $$C=\frac{T\left(T-1\right)B}{\chi }$$

**Proof.**  Because the capacity of the ISL is *B* and there are a total of χ flows that need to be transmitted through a bottleneck link, the magnitude of the maximum achievable flow is $\xi =\frac{B}{\chi }$. Substituting this into Equation (8) yields Equation (9). □

Notably, χ is closely related to the topology of the ML-LEO. In Section 2.3, we classify ML-LEO topologies into two cases: L=2 (Case 1) and L>2 (Case 2). In order to explore the location of bottleneck link and the corresponding χ, the analysis of χ is also divided into these two cases. In addition, the bottleneck links in ML-LEOs are always the ISLs connected to key satellites. This is because each key satellite on the *i*-th layer carries not only flows where both the source and destination satellites are on the *i*-th layer, but also all flows where the source and destination satellites are on different layers relative to the *i*-th layer. Furthermore, the intra-layer bottleneck links are either the intra-orbit or inter-orbit ISLs connected to key satellites. To summarize, the core idea of our capacity analysis is as follows: we analyze the number of flows passing through the intra-orbit ISLs $\chi_{ao}\left(i\right)$, inter-orbit ISLs $\chi_{eo}\left(i\right)$, and inter-layer ISLs $\chi_{el}\left(i\right)$ of key satellites on the *i*-th layer, and take the maximum value as χ, i.e.,

(10) $$\chi =\max_{i}\left\{\max\left[\chi_{ao}\left(i\right),\;\chi_{eo}\left(i\right),\;\chi_{el}\left(i\right)\right]\right\},\;i\in \left\{1,2,\ldots ,L\right\}.$$

### 3.2. Capacity Analysis for ML-LEOs of Two Cases

In this section, we first focus on Case 1 and derive the throughput capacity expression for this scenario. We then present a detailed analysis of $\chi_{ao}\left(i\right)$ and $\chi_{el}\left(i\right)$. Due to topological symmetry, the analysis for $\chi_{eo}\left(i\right)$ is identical to that for $\chi_{ao}\left(i\right)$ in terms of analytical logic. For Case 2, we first present the capacity expression in Section 3.2.2, followed by the key analysis steps.

#### 3.2.1. Analysis of Case 1

**Theorem** **2.** *The capacity of two-layer ML-LEOs is*

(11) $$C=\left\{\begin{array}{cc} \frac{T^{2}B}{\frac{N_{i}M_{i}^{2}}{8}+\frac{T_{1}T_{2}}{4n_{\left(1,2\right)}}+\frac{1}{8N_{i}}T_{1}T_{2}\left(1-\frac{2}{b_{\left(1,2\right)}}\right)} & \chi =\chi_{ao}^{1}\left(i\right)\\ \frac{T^{2}B}{\frac{N_{i}^{2}M_{i}}{8}+\frac{T_{1}T_{2}}{4n_{\left(1,2\right)}}+\frac{1}{8M_{i}}T_{1}T_{2}\left(1-\frac{2}{a_{\left(1,2\right)}}\right)} & \chi =\chi_{eo}^{1}\left(i\right)\\ \frac{T^{2}Bn_{\left(1,2\right)}}{T_{1}T_{2}} & \chi =\chi_{el}^{1}\left(i\right) \end{array}\right..$$

*Here, $\chi_{ao}^{1}\left(i\right)$ denotes the number of flows passing through the intra-orbit ISLs of key satellites on the i-th layer for Case 1. In addition, $\chi_{eo}^{1}\left(i\right)$ and $\chi_{el}^{1}\left(i\right)$ represent the number of flows through the inter-orbit ISLs and the number of flows through the inter-layer ISLs of the key satellite on the i-th layer in Case 1.*

*For Case 1, $\chi_{ao}^{1}\left(i\right)$ consists of two components as follows, i.e., $\chi_{ao}^{1}\left(i\right)=\chi_{ao1}^{1}\left(i\right)+\chi_{ao2}^{1}\left(i\right)$.*

1. *$\chi_{ao1}^{1}\left(i\right)$: flows where both the source and destination satellites are on the i-th layer.*
2. *$\chi_{ao2}^{1}\left(i\right)$: flows where the source satellites are on the i-th layer and the destination satellites are on the adjacent layer.*

*Among them, $\chi_{ao1}^{1}\left(i\right)=\frac{N_{i}M_{i}^{2}}{8}$. The relevant proofs are provided in Appendix A.*

*For $\chi_{ao2}^{1}\left(i\right)$, we further decompose it into two sub-components for analysis as follows, i.e., $\chi_{ao2}^{1}\left(i\right)=\chi_{ao21}^{1}\left(i\right)+\chi_{ao22}^{1}\left(i\right)$.*

1. *$\chi_{ao21}^{1}\left(i\right)$: flows that must pass through the intra-orbit ISLs of key satellites.*
2. *$\chi_{ao22}^{1}\left(i\right)$: flows that may pass through the intra-orbit ISLs of key satellites.*

*Among them, $\chi_{ao21}^{1}\left(i\right)=\frac{N_{1}M_{1}}{4}\frac{N_{2}M_{2}}{n_{\left(1,2\right)}}$ and $\chi_{ao22}^{1}\left(i\right)=\frac{1}{8N_{i}}T_{1}T_{2}\left(1-\frac{2}{b_{\left(1,2\right)}}\right)$. The relevant proofs are provided in Appendices Appendix B and Appendix C, respectively.*

*Finally, we can obtain*

(12) $$\chi_{ao}^{1}\left(i\right)=\frac{N_{i}M_{i}^{2}}{8}+\frac{TT_{2}}{4n_{\left(1,2\right)}}+\frac{1}{8N_{i}}T_{1}T_{2}\left(1-\frac{2}{b_{\left(1,2\right)}}\right).$$

*Following the analysis of intra-layer ISLs, we next derive the number of flows passing through inter-layer ISLs for two-layer ML-LEOs. The number of flows passing through each inter-layer ISL of a two-layer ML-LEO is*

(13) $$\chi_{el}^{1}=\frac{T_{1}T_{2}}{n_{\left(1,2\right)}}.$$

**Proof.**  All flows whose source satellites and destination satellites are on the different layer must pass through inter-layer ISLs. Thus, a total of $T_{1}T_{2}$ inter-layer flows are transmitted through $n_{\left(1,2\right)}$ inter-layer ISLs. □

In summary, the above analysis leads to Theorem 2.

#### 3.2.2. Analysis of Case 2

**Theorem** **3.** *The throughput capacity of ML-LEOs with L>2 (Case 2) takes a form analogous to (11):*

(14) $$C=\left\{\begin{array}{cc} T^{2}B/\chi_{ao}^{2}\left(i\right) & \chi =\chi_{ao}^{2}\left(i\right)\\ T^{2}B/\chi_{eo}^{2}\left(i\right) & \chi =\chi_{eo}^{2}\left(i\right)\\ T^{2}B/\chi_{el}^{2}\left(i\right) & \chi =\chi_{el}^{2}\left(i\right) \end{array}\right..$$

*Here, $\chi_{ao}^{2}\left(i\right)$ denotes the number of flows passing through the intra-orbit ISLs of key satellites on the i-th layer for Case 2. In addition, $\chi_{eo}^{2}\left(i\right)$ and $\chi_{el}^{2}\left(i\right)$ represent the number of flows through the inter-orbit ISL and the number of flows through the inter-layer ISL of the key satellite on the i-th layer in Case 2.*

*The analysis process for Case 2 is as follows. As mentioned previously, the i-th layer (where 1<i<L) contains two types of key satellites: $\kappa_{\left(i,i-1\right)}$ and $\kappa_{\left(i,i+1\right)}$. Bottlenecks can only occur at four locations: the intra-orbit and inter-orbit ISLs of $\kappa_{\left(i,i-1\right)}$, and the intra-orbit and inter-orbit ISLs of $\kappa_{\left(i,i+1\right)}$. In fact, due to the uniform distribution of inter-layer ISLs, these four types of ISLs can be grouped into two categories:*

*1.*  *The intra-orbit ISLs of $\kappa_{\left(i,i-1\right)}$ or $\kappa_{\left(i,i+1\right)}$.*
*2.*  *The inter-orbit ISLs of $\kappa_{\left(i,i-1\right)}$ or $\kappa_{\left(i,i+1\right)}$.*

*We take $\chi_{ao}^{2}\left(i\right)$ as an example to present the detailed analysis and the analysis for $\chi_{eo}^{2}\left(i\right)$ is identical due to topological symmetry. For Case 2, the total number of flows passing through all intra-orbit ISLs on the same orbital row of key satellites on the i-th layer consists of seven components, i.e., $\chi_{\mathrm{aosum}}^{2}\left(i\right)=\sum_{j=1}^{7}\chi_{\mathrm{aoj}}^{2}\left(i\right)$. We first present the expressions for these seven components and the relevant proofs are provided in Appendix D.*

*Part I: The number of flows $\chi_{ao1}^{2}\left(i\right)$ whose source and destination satellites are all on the i-th layer, where*

(15) $$\chi_{ao1}^{2}\left(i\right)=\frac{N_{i}^{2}M_{i}^{2}}{8}.$$

*Part II: The number of flows $\chi_{ao2}^{2}\left(i\right)$ whose source satellites are on the lower layer and the destination satellites are on the upper layer, where*

(16) $$\chi_{ao2}^{2}\left(i\right)=a_{\left(i,i+1\right)}\frac{\sum_{t=1}^{i-1}T_{t}\sum_{j=i+1}^{L}T_{j}}{4n_{\left(i,i+1\right)}}\left(1-\alpha_{\left(i,i+1\right)}\right).$$

*Part III: The number of flows $\chi_{ao3}^{2}\left(i\right)$ whose source satellites are on the upper layer, and the destination satellites are on the lower layer, where*

(17) $$\chi_{ao3}^{2}\left(i\right)=a_{\left(i,i-1\right)}\frac{\sum_{t=1}^{i-1}T_{t}\sum_{j=i+1}^{L}T_{j}}{4n_{\left(i,i-1\right)}}\left(1-\alpha_{\left(i,i-1\right)}\right).$$

*Part IV: The number of flows $\chi_{ao4}^{2}\left(i\right)$ whose source satellites are on the i-th layer and the destination satellites are on the upper layer, where*

(18) $$\chi_{ao4}^{2}\left(i\right)=\frac{N_{i}M_{i}\sum_{t=i+1}^{L}T_{t}}{8}.$$

*Part V: The number of flows $\chi_{ao5}^{2}\left(i\right)$ whose source satellites are on the i-th layer and the destination satellites are on the lower layer, where*

(19) $$\chi_{ao5}^{2}\left(i\right)=\frac{N_{i}M_{i}\sum_{t=1}^{i-1}T_{t}}{8}.$$

*Part VI: The number of flows $\chi_{ao6}^{2}\left(i\right)$ whose source satellites are on the upper layer and the destination satellites are on the i-th layer, where*

(20) $$\chi_{ao6}^{2}\left(i\right)=\frac{a_{\left(i,i+1\right)}\sum_{t=i+1}^{L}T_{t}}{4}\left(\frac{T_{i}}{n_{\left(i,i+1\right)}}-1\right).$$

*Part VII: The number of flows $\chi_{ao7}^{2}\left(i\right)$ whose source satellites are on the lower layer and the destination satellites are on the i-th layer, where*

(21) $$\chi_{ao7}^{2}\left(i\right)=\frac{a_{\left(i,i-1\right)}\sum_{t=1}^{i-1}T_{t}}{4}\left(\frac{T_{i}}{n_{\left(i,i-1\right)}}-1\right).$$

*Next, we consider the distribution of these flows across these ISLs. An even distribution is optimal because it minimizes the value of $\chi_{ao}^{2}\left(i\right)$. However, this approach has two limitations. First, the derived capacity is not a tight bound, and the even distribution may not be achievable for all topologies. Second, some flows must pass through specific ISLs and thus cannot be evenly distributed. However, considering all these factors will result in an overcomplicated analysis with numerous scenarios. In addition, the even distribution can already yield a relatively reasonable result and the subsequent simulations have also verified this. Therefore, we can distribute $\chi_{aosum}^{2}\left(i\right)$ evenly to each ISL on the same row to obtain $\chi_{ao}^{2}\left(i\right)$, namely $\chi_{ao}^{2}\left(i\right)=\frac{\chi_{aosum}^{2}\left(i\right)}{N_{i}}$.*

*The number of flows through inter-layer ISLs between the i-th layer and the $\left(i+1\right)$-th layer can be obtained by Equation (22):*

(22) $$\chi_{el}^{2}\left(i\right)=\left(\sum_{j=1}^{i}T_{j}\sum_{k=i+1}^{L}T_{k}\right)/n_{\left(i,i+1\right)}$$

*The derivation is similar to that for Equation (13). In summary, we can obtain the capacity for Case 2 as Theorem 3.*

## 4. Optimal Parameter Configuration

The previous analysis has shown the capacity of ML-LEOs. However, what attracts us more is how to achieve maximum capacity with the same number of satellites. Optimizing the parameter configuration of ML-LEOs can help us achieve maximum capacity with minimum satellite count. In this section, we take the ML-LEOs with the identical intra-layer topology of each layer and $n_{\left(i,i-1\right)}=n_{\left(i,i+1\right)}=n$, $\alpha_{\left(i,i-1\right)}=\alpha_{\left(i,i+1\right)}=1$ as an example. Under the constraint that the number of total satellites *T* is constant, we analyze the following optimization problems:

(1) The optimal number of layers $L_{op}$ when inter-layer ISLs are fully deployed.
(2) The optimal number of inter-layer ISLs $n_{op}$ when the number of layers and the topology of each layer are fixed.
(3) The optimal topology $N_{iop}\times M_{iop}$ of each layer when the number of layers is constant and the number of inter-layer ISLs is maximized, i.e., $n=N_{i}M_{i}$. Here we also compare the capacity of SL-LEOs and ML-LEOs of the same scale to verify the advantages of multi-layer configuration in capacity.

### 4.1. The Optimal Number of Layers $L_{op}$

When studying the relationship between the capacity and the topology of ML-LEOs, a question will inevitably arise: when the number of satellites is a constant, whether the more layers, the greater the capacity. For the analysis of the optimal number of layers $L_{op}$, the influence of the intra-layer ISL rate $B_{xy}$ and the inter-layer ISL rate $B_{z}$ on capacity also needs to be considered:

(23) $$\chi =\left\{\begin{array}{cc} \frac{T\left(T-1\right)}{8N_{i}L} & \chi =\chi_{ao}\left(i\right)\\ \frac{T^{2}}{4n} & \chi =\chi_{el}\left(i\right) \end{array}\right.$$

(24) $$C=\left\{\begin{array}{cc} 8N_{i}LB_{xy} & \chi =\chi_{ao}\left(i\right)\\ 4nB_{z} & \chi =\chi_{el}\left(i\right) \end{array}\right.$$

Equation (23) and Equation (24) show χ and the capacity in this scenario, respectively. The analysis reveals that there are two situations for the relationship between *C* and *L*. The first one is that the bottleneck is exclusively the inter-layer ISLs or the intra-layer ISLs, which corresponds to infinite $B_{xy}$ or infinite $B_{z}$, respectively. However, practical ISL data rates are finite; we therefore exclude this idealized case from further analysis. The second is that when *L* is less than $L^{*}$, the threshold of the number of layers, the bottleneck is exclusively the intra-layer ISLs and the capacity will monotonically increase with the number of layers. However, when *L* exceeds $L^{*}$, the bottleneck will shift to the inter-layer ISLs and the capacity begins to decline gradually. This inflection point $L^{*}$, coinciding with the optimal layer count $L_{op}$, is given by

(25) $$L_{op}=\frac{nB_{z}}{2NB_{xy}}.$$

It is worth noting that Equation (25) also indicates the condition that the ISL rate should meet to achieve the maximum capacity. If $B_{xy}=B_{z}$ and *n* reach the maximum, the optimal number of layers is $L_{op}=\frac{M_{i}}{2}$.

### 4.2. The Optimal Configuration of Inter-Layer Links $n_{op}$

Equation (26) shows the number of flows passing through the bottleneck link in this scenario:

(26) $$\chi =\left\{\begin{array}{cc} \frac{1}{8}N_{i}M_{i}^{2}\left(1+\left(L-1\right)\left(\frac{2}{n}+1\right)\right) & n\geq n^{*}\\ \frac{T^{2}}{4n} & n<n^{*} \end{array}\right..$$

Equation (26) reveals that when *n* is greater than the threshold $n^{*}$, the intra-layer ISLs are the bottleneck. For large *n*, $\frac{2}{n}$ is approximately 0 for ML-LEOs. In this case, χ can be approximated as $\frac{1}{8}N_{i}M_{i}^{2}L$, coinciding with the maximum *n* case. When *n* is less than the threshold $n^{*}$, the inter-layer ISLs will be the bottleneck and χ increases rapidly with the decrease of *n*. Because *C* decreases monotonically with the increase in χ, $n^{*}$ is also the optimal value of inter-layer ISLs. According to Equation (26), the approximation of $n_{op}$ is

(27) $$n_{op}=2N_{i}L.$$

The capacity *C* at this time is approximately equal to $8N_{i}LB$, which is the capacity when $n=N_{i}M_{i}$.

### 4.3. The Optimal Inter-Layer Topology $N_{iop}\times M_{iop}$

According to the analysis in Section 3.2.2, we get the capacity in this scene as

(28) $$C=\left\{\begin{array}{cc} 8N_{i}LB & N_{i}\leq M_{i}\\ 8M_{i}LB & N_{i}>M_{i} \end{array}\right.,$$

and it can be re-expressed as

(29) $$C=\left\{\begin{array}{cc} 8N_{i}LB & N_{i}\leq M_{i}\\ 8\frac{T_{i}}{N_{i}}LB & N_{i}>M_{i} \end{array}\right..$$

According to Equation (29), if we keep $M_{i}$ as a constant, *C* will first increase and then decrease with the increase in $N_{i}$, and reaches the maximum $8\sqrt{TL}B$ when $N_{i}=M_{i}$. The same trend also holds for $M_{i}.$ The reason is that when $N_{i}\leq M_{i}$, there will be more flows through the intra-orbit ISLs, so the intra-orbit ISLs will be the bottleneck links. If we increase $N_{i}$, the flows through the intra-orbit ISLs will gradually transfer to inter-orbit ISLs, which have not yet reached saturation, so the capacity will increase until $N_{i}=M_{i}$. At this point, flows will evenly pass through the intra-orbit ISLs and the inter-orbit ISLs. If we continue increasing $N_{i}$, that is $N_{i}>M_{i}$, at this point the inter-orbit ISLs are the bottleneck links, so the capacity will decrease.

The capacity expression of SL-LEOs is $C=\left\{\begin{array}{cc} 8NB & N\leq M\\ 8MB & N>M \end{array}\right.$, where *N* and *M* represent the number of orbits and satellites per orbit, respectively [22]. Obviously, when N=M, *C* reaches $8\sqrt{T}B$, which is the maximum. Comparing $8\sqrt{TL}B$ and $8\sqrt{T}B$, ML-LEOs achieve a capacity gain of $\sqrt{L}$ relative to SL-LEOs (neglecting the regime where inter-layer ISLs become bottlenecke for large *L*). Thus, the advantages of multi-layer networks in terms of capacity have been proved.

Finally, we can conclude that in order to achieve maximum capacity, the number of layers should satisfy $L_{op}=\frac{M_{i}}{2}$, and the intra-layer topology should be $N_{iop}=M_{iop}$. In addition, the optimal configuration of inter-layer ISLs is $n_{op}=2N_{i}L$.

## 5. Simulation

In this section, we perform the capacity simulations for ML-LEOs fixed total satellite count *T* but different topologies based on STK 11 (Satellite Tool Kit) and MATLAB 2018b (Matrix Laboratory). In the following simulation scenarios, the total number of satellites is fixed at approximately 1000. This scale is selected to validate the theoretical scaling laws derived in this paper with manageable computational complexity. In addition, the total number of satellites is not strictly fixed at 1000 since some topologies with exactly 1000 satellites yield capacity below meaningful comparison thresholds. The inclination angles are set within $30^{\circ }$–$60^{\circ }$, covering typical deployment strategies for global-coverage constellations serving major communication markets. In addition, to ensure a fair comparison, the optimal intra-layer topology is adopted for each layer.

First, in order to investigate the impact of the number of layers *L* on the capacity and verify the optimal $L_{op}$, the parameters are shown in Table 2. We maintain the total satellite count at approximately 1000 and increase the number of layers to observe the relationship between the number of layers and the capacity.

Second, to explore the impact of the number of inter-layer ISLs on system capacity and verify the optimal configuration $n_{op}$, we conduct another set of simulations, whose scenario parameters are presented in Table 3. We keep the total number of satellites, the intra-layer topology, and other relevant parameters unchanged, and only adjust the number of inter-layer ISLs between each pair of layers.

Third, to explore the impact of intra-layer topology on system capacity, we set up two groups of scenarios. For the first group, the scenario parameters except for $M_{i}$ are shown in Table 4. We keep the number of orbits constant and observe the impact of the number of satellites per orbit on capacity. For a fair comparison, the number of inter-layer ISLs *n* is set to the maximum value. Due to the symmetry of the topology, the impact of the number of orbits on capacity is the same as that of the number of satellites per orbit. The scenario parameters of the second group are shown in Table 5. We consider three-layer ML-LEOs with approximately 1000 satellites in total and observe the impact of intra-layer topology on capacity. It should be noted that due to the symmetry of ML-LEOs, additional scenarios with $N_{i}>M_{i}$ are unnecessary.

Finally, to compare the advantages of ML-LEOs over SL-LEOs, we present several SL-LEOs and their corresponding capacity in Table 6. These SL-LEOs have different topologies but the same total number of satellites as those in Table 5. Similarly, the optimal topology is also selected for these SL-LEO networks. The height in Table 6 is selected to match the middle layer of the ML-LEO scenarios in Table 5 for fair comparison. Since SL-LEOs do not involve inter-layer ISLs or multiple orbital layers, a single height is sufficient to characterize their capacity under the 2D-torus topology.

**Figure 5:**
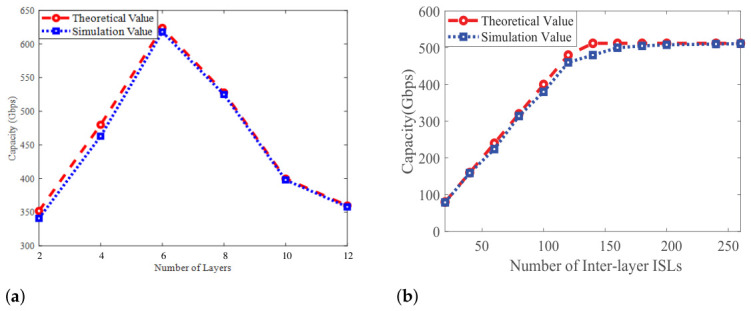
Relationship between the capacity and *L* and *n*. (**a**) The impact of the number of layers. (**b**) The impact of the number of inter-layer ISLs.

Figure 5a illustrates the relationship between system capacity and the number of layers *L*. It can be observed that when the total number of satellites is kept constant, the capacity first gradually increases and then decreases as the number of layers increases. This phenomenon can be explained as follows: when L⩽6, the number of satellites on each layer is sufficient to establish an adequate number of inter-layer ISLs, making intra-layer ISLs the bottleneck of the system. At this point, increasing *L* redirects more traffic originally intended for intra-layer ISLs to inter-layer ISLs, which remain unsaturated, thereby improving the capacity. However, when L>6, the number of satellites per layer decreases, which limits the number of inter-layer ISLs and causes inter-layer ISLs to become the new bottleneck. In this case, a larger *L* leads to fewer inter-layer ISLs and a heavier traffic burden on these ISLs, resulting in lower system capacity.

Figure 5b illustrates the trend of system capacity with respect to the number of inter-layer ISLs *n*. It can be observed that when the number of inter-layer ISLs is less than the threshold of 120, the capacity increases linearly with the increase in *n*; however, when *n* exceeds 120, the change in capacity tends to stabilize. This phenomenon can be explained as follows: initially, the bottleneck of the system is the inter-layer ISLs, so *n* exerts a significant impact on the system capacity. When *n* exceeds 120, the bottleneck shifts to the intra-layer ISLs. At this point, as *n* increases, the system capacity gradually approaches $8N_{i}L$, leading to a gradual stabilization of the capacity trend.

**Figure 6:**
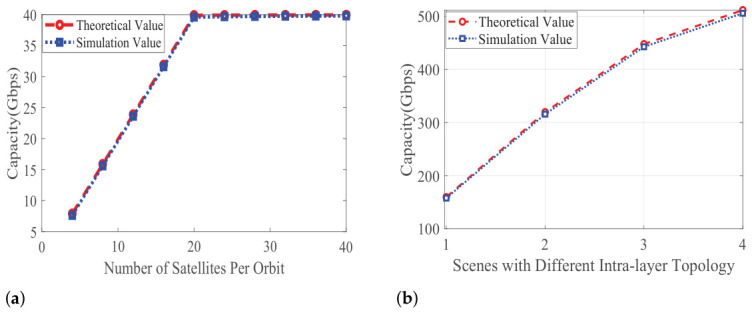
Relationship between the capacity and $M_{i}$ and intra-layer topology. (**a**) The impact of the number of satellites per orbit. (**b**) The impact of the intra-layer topology.

Figure 6a illustrates the influence of the number of satellites per orbit on capacity. It can be observed that when the number of satellites per orbit is less than the number of orbits, the capacity increases monotonically with the increase in the number of satellites per orbit. However, when the number of satellites per orbit exceeds the number of orbits, increasing the number of satellites per orbit has no significant impact on the capacity. This phenomenon can be explained as follows: when the number of satellites per orbit is less than the number of orbits, the bottleneck of the system is the inter-orbit ISLs, and the maximum capacity is approximately $C=8M_{i}LB$. Increasing the number of satellites per orbit will significantly improve the system capacity. When the number of satellites per orbit exceeds the number of orbits, the bottleneck shifts from inter-orbit ISLs to intra-orbit ISLs. At this point, the approximate maximum capacity becomes $C=8N_{i}LB$, so increasing the number of satellites per orbit has little effect on the capacity. Thus, the optimal topology satisfies $N_{i}=M_{i}$.

Figure 6b illustrates the relationship between system capacity and intra-layer topology under the condition that the total number of satellites and the number of layers are kept constant. It can be observed that different intra-layer topologies have a significant impact on system capacity, and the closer the number of orbits is to the number of satellites per orbit, the greater the capacity. This is because the traffic flows through the inter-orbit ISLs and intra-orbit ISLs for key satellites become more balanced. Consequently, $\chi_{max}$ becomes smaller, leading to an increase in system capacity.

**Figure 7:**
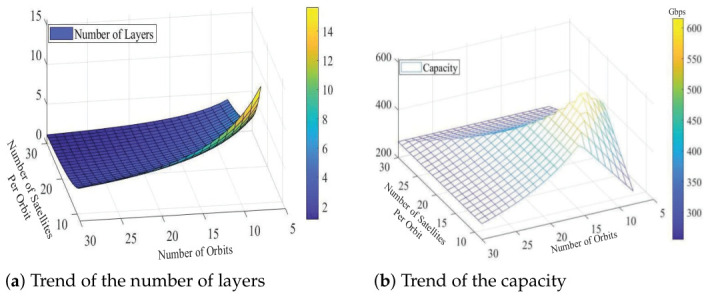
Capacity trend diagram.

Figure 7a shows the relationship between layer count and intra-layer topology. Figure 7b shows the corresponding capacity surface. We maintain total satellite count at approximately 1000, with $N_{i},M_{i}\in \left[8,30\right]$. Because $T=L\cdot N_{i}\cdot M_{i}$ is fixed, *L* decreases as $N_{i}$ and $M_{i}$ increase. Figure 7b reveals that capacity increases monotonically as $N_{i}$ and $M_{i}$ converge, peaking at $N_{i}=M_{i}$. Capacity also exhibits non-monotonic dependence on *L*: for small *L*, intra-layer ISLs are the bottleneck, and increasing *L* reduces per-ISL load, improving capacity; for large *L*, inter-layer ISLs become the bottleneck, and additional layers aggravate cross-layer congestion, reducing capacity. Note that although *L* takes integer values only, we connect adjacent discrete points with lines in Figure 7 to improve readability. Isolated points or bar charts would obscure the visual trends and make coordinate identification more difficult.

Table 6 presents SL-LEO capacities with approximately 1000 satellites. Compared to the ML-LEO results in Figure 6b, ML-LEOs achieve substantially higher capacity than SL-LEOs. Note that both configurations employ the optimal intra-layer topology. Under this condition, ML-LEO advantages are evident. However, if the ML-LEO adopts a non-optimal topology (e.g., $N_{i}\neq M_{i}$) while the SL-LEO remains optimally configured, the SL-LEO may outperform the ML-LEO.

The relative error between theoretical and simulation results is within 5% for all scenarios. The maximum discrepancy appears at L=4 in Figure 5a. The theoretical capacity is 480 Gbps and the simulation capacity is 463 Gbps, with a relative error of 3.5%. This level of error is acceptable because the simulation captures several practical effects. First is dynamic ISL visibility due to satellite motion, which temporarily reduces available ISLs and increases bottleneck load. Secend is discrete routing effects where multiple minimum-hop paths exist and the simulation randomly selects among them, preventing perfectly balanced flow distribution.

## 6. Conclusions

In this paper, we present a comprehensive analysis of how ML-LEO configuration affects throughput capacity. We first define the inter-layer coupling coefficient to characterize inter-layer ISL distribution and formalize the ML-LEO topological model. Based on this framework, we distinguish between edge cases (L=2) and general configurations (L>2), deriving capacity expressions for each regime by analyzing bottleneck link loads. We demonstrate that multi-layer architectures enable $\sqrt{L}$ capacity scaling relative to SL-LEOs under uniform layer configuration ($N_{i}=M_{i}$). Furthermore, we establish optimal parameter configurations: $N_{op}=M_{op}$, $L_{op}=M_{op}/2$ (under fixed total satellite count), and $n_{op}=2N_{op}L$ per adjacent layer pair. Extensive simulations examine capacity dependence on key topological parameters—including layer count, satellites per orbit, and inter-layer ISL density—validating our theoretical analysis. These results evaluate and guide constellation configuration optimization.

## Figures and Tables

**Table 1:** Summary of notations.

Symbol	Meaning
*T*	Total number of satellites
$T_{i}$	Number of satellites on the *i*-th layer
$N_{i}$	Number of orbits of the *i*-th layer
$M_{i}$	Number of satellites per orbit of the *i*-th layer
*L*	Number of layers
$n_{\left(i,i+1\right)}$	Number of inter-layer ISLs between the *i*-th and the $\left(i+1\right)$-th layer
$\alpha_{\left(i,i+1\right)}$	Inter-layer coupling coefficient between the *i*-th layer and the $\left(i+1\right)$-th layer
ξ	Size of the maximum achievable flow of the network
χ	Number of flows through bottleneck links
$\chi_{ao}\left(i\right)$	Number of flows through the intra-orbit ISL of the key satellite on the *i*-th layer
$\chi_{eo}\left(i\right)$	Number of flows through the inter-orbit ISl of the key satellite on the *i*-th layer
$\chi_{al}\left(i\right)$	Number of flows through the inter-layer ISLs between the *i*-th and the $\left(i+1\right)$-th layer
*C*	Throughput capacity

**Table 2:** Simulation scenes for ML-LEOs with different *L*.

*T*	$N_{i}$	$M_{i}$	*n*	*L*	Inclination ${(}^{\circ })$	$B\;\left(\mathrm{Gbps}\right)$
1056	22	24	528	2	60	1
1020	15	17	255	4		
1014	13	13	169	6		
1056	11	12	132	8		
1000	10	10	100	10		
1080	9	10	90	12		

**Table 3:** Simulation scenes for ML-LEOs with different *n*.

$N_{i}$	$M_{i}$	*L*	Height (km)	Inclination ${(}^{\circ })$	$B\;\left(\mathrm{Gbps}\right)$
16	16	4	500 600 700 800	60	1

**Table 4:** Simulation scenarios for ML-LEOs with different $M_{i}$.

$N_{i}$	*L*	Height (km)	Inclination ${(}^{\circ })$	$B\;\left(\mathrm{Gbps}\right)$
20	4	328/334/345/350	42/48/53/53	1

**Table 5:** Simulation scenarios for ML-LEOs with different intra-layer topology.

	Scene 1	Scene 2	Scene 3	Scene 4
*T*	1000	1000	1064	1024
Height (km)	335/340/345/350			
Inclination ${(}^{\circ })$	30/40/53/53			
*B* (Gbps)	1			
*L*	4			
$N_{i}$	5	10	14	16
$M_{i}$	50	25	19	16

**Table 6:** Simulation scenarios for SL-LEOs.

	Scene 1	Scene 2	Scene 3	Scene 4
*T*	1000	1020	1000	1020
*N*	10	15	20	30
*M*	100	68	50	34
Height (km)	340			
Inclination ${(}^{\circ })$	40			
*B* (Gbps)	1			
$C_{T}$ (Gbps)	79.92	119.88	159.84	247.88
$C_{S}$ (Gbps)	77.52	115.64	154.23	240.95

**Figure A1:**
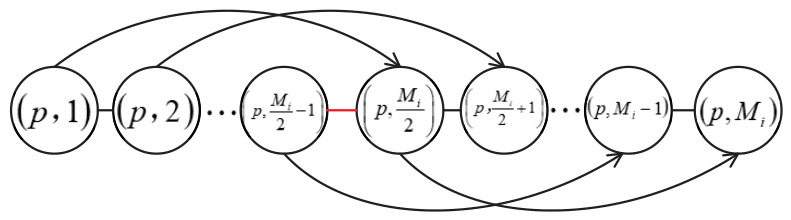
Illustration of $\chi_{ao1}^{1}\left(i\right)$.

**Figure A2:**
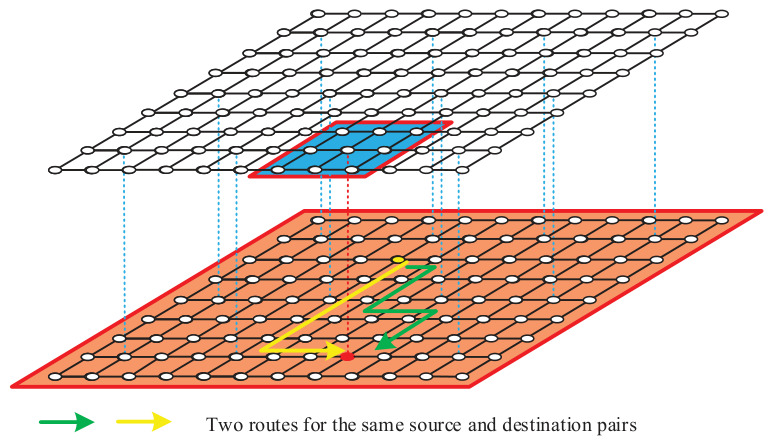
Illustration of $\chi_{ao2}^{1}\left(i\right)$ and “Detor”.

**Figure A3:**
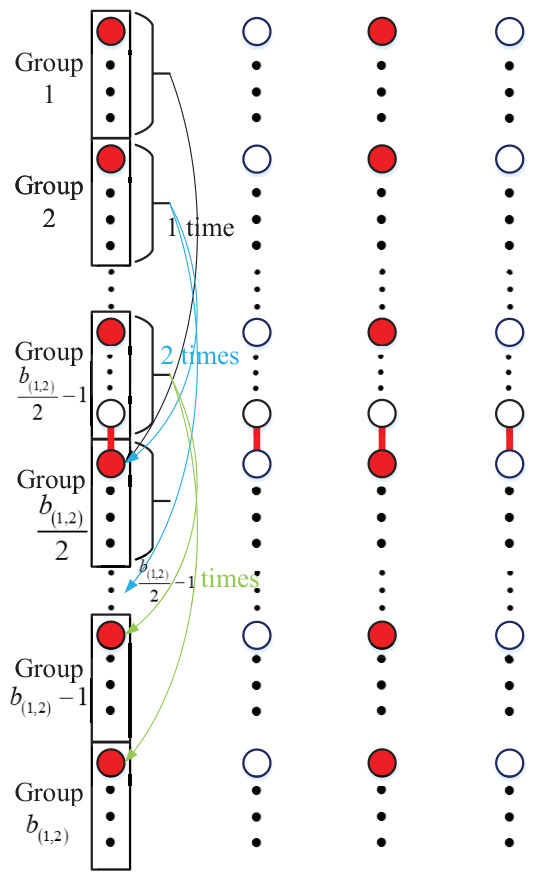
Analysis illustration of $\chi_{22}\left(i\right)$.

## Data Availability

The data presented in this study are available in the article. The raw data supporting the reported results are available from the corresponding author upon reasonable request.

## Appendix A. Proof for $\chi_{ao1}^{1}\left(i\right)$

Since the source and destination satellites are co-located on the same layer, no inter-layer ISLs are involved. Accordingly, key satellite ISLs experience identical load conditions as ordinary satellite ISLs. The analysis schematic for $\chi_{ao1}^{1}\left(i\right)$ is presented in Figure A1. For simplicity, the layer index *i* is omitted hereafter, and the diagram is oriented vertically.

For any intra-orbit ISL $\left(p,q\right)\to \left(p,q+1\;\mathrm{mod}\;M_{i}\right)$, this ISL carries $\frac{M_{i}}{2}-0.5$ flows when node (p,q) transmits to other satellites on the same orbit. Specifically, flows destined for $\left(p,q+1\;\mathrm{mod}\;M_{i}\right)$, $\left(p,q+2\;\mathrm{mod}\;M_{i}\right)$, …, $\left(p,q+\frac{M_{i}}{2}-1\;\mathrm{mod}\;M_{i}\right)$ each pass through this ISL once. For the antipodal node $\left(p,q+\frac{M_{i}}{2}\;\mathrm{mod}\;M_{i}\right)$, two equidistant paths exist; with traffic split equally, this contributes an expected additional load of 0.5. Thus, the total number of flows through any intra-orbit ISL on layer *i* is

$$N_{i}\sum_{j=1}^{M_{i}/2}\left(j-0.5\right)=\frac{N_{i}M_{i}^{2}}{8},\;\mathrm{for}\;\mathrm{even}\;M_{i}.$$

## Appendix B. Proof for $\chi_{ao21}^{1}\left(i\right)$

Note that these flows exhibit a distinctive property for $\chi_{ao2}^{1}\left(i\right)$ under the routing in Section 2.4. Taking layer 1 as an example, when all layer-1 satellites serve as sources sending flows to all layer-2 satellites as destinations, these flows first uniformly converge to all key satellites on layer 1, then traverse inter-layer ISLs to layer 2. However, flows converging to a given key satellite on layer 1 can only reach specific subsets of layer-2 satellites. Figure A2 illustrates the source-destination mapping for flows converging to a key satellite on layer 1. Among the flows counted in $\chi_{ao2}\left(1\right)$, those converging to the red key satellite (Figure A2) originate from all layer-1 satellites (shaded light red) but terminate only at the subset of layer-2 satellites shaded blue. The red solid line indicates this source-destination mapping. These flows constitute $\chi_{ao21}^{1}\left(i\right)$, as they must ultimately converge to key satellites regardless of routing path.

From these properties, we obtain

$$\chi_{ao21}^{1}\left(i\right)=\frac{N_{1}M_{1}}{4}\cdot \frac{N_{2}M_{2}}{n_{\left(1,2\right)}}.$$

Each key satellite serves $\frac{N_{2}M_{2}}{n_{\left(1,2\right)}}$ layer-2 satellites, and $\frac{N_{1}M_{1}}{4}$ key satellites participate in this traffic aggregation (under uniform distribution with α=1). All layer-1 satellites transmit to all layer-2 satellites via the intra-layer ISLs that converge to these key satellites.

## Appendix C. Proof for $\chi_{ao22}^{1}\left(i\right)$

$\chi_{ao22}^{1}\left(i\right)$ arises from the following scenario. When a layer-1 satellite transmits flows to layer-2 satellites via an intra-orbit ISL toward a key satellite, these flows may traverse intra-orbit ISLs of other key satellites while traveling toward their designated relay. This explains why some flows pass through intra-orbit ISLs of multiple key satellites. Figure A2 illustrates this phenomenon. When the yellow node serves as source for flows destined for blue-marked layer-2 satellites via the red key satellite (its designated relay), at least two routing options exist: the yellow path and the green path. Along the green path, flows traverse only the red key satellite. In contrast, the yellow path traverses two additional key satellites. Although multiple available paths exist with varying key satellite sequences, all such flows converge at their designated relay.

In other words, any flow traversing intra-orbit ISLs of key satellites within the same orbital row necessarily passes through the intra-orbit ISLs of a specific satellite row. Figure A3 depicts this mechanism; non-key ISLs are omitted, and only key satellites and numbered member satellites are retained. All flows from Groups 1 to $\frac{b_{\left(1,2\right)}}{2}-1$ targeting key satellites of Group $\frac{b_{\left(1,2\right)}}{2}$ in each orbit necessarily pass through the marked ISLs. The subsequent analysis proceeds in two steps:

1. Focus on the sum of the flows $\chi_{ao22sum}^{1}$ through all intra-orbit ISLs in the same row of key satellites (highlighted in red in Figure A3).
2. Discuss how to assign these flows.

First, we select one orbit containing key satellites and analyze flows with both source and key-relay satellites on this orbit, specifically through intra-orbit ISLs between Group $\frac{b_{\left(1,2\right)}}{2}-1$ and Group $\frac{b_{\left(1,2\right)}}{2}$. Due to the cyclic symmetry of the 2-D torus, any such orbit is equivalent. The $b_{\left(1,2\right)}$ key satellites on each orbit partition the $M_{i}$ satellites into $b_{\left(1,2\right)}$ groups of $\frac{M_{i}}{b_{\left(1,2\right)}}$ satellites each.

Flows from Group 1 traverse this ISL exactly once, as they must reach key-relay satellites in Group $\frac{b_{\left(1,2\right)}}{2}+1$. Flows from Group 2 traverse it twice, reaching Groups $\frac{b_{\left(1,2\right)}}{2}+1$ and $\frac{b_{\left(1,2\right)}}{2}+2$. Continuing this pattern, flows from Group $\frac{b_{\left(1,2\right)}}{2}$ traverse it $\frac{b_{\left(1,2\right)}}{2}-1$ times, reaching Groups $\frac{b_{\left(1,2\right)}}{2}+1$ through $b_{\left(1,2\right)}-1$. Thus, when source and key-relay satellites are co-orbital, the total flows through this ISL are

$$\frac{M_{i}}{b_{\left(1,2\right)}}\left(1+2+\cdots +\frac{b_{\left(1,2\right)}}{2}-1\right)=\frac{1}{8}M_{i}b_{\left(1,2\right)}\left(b_{\left(1,2\right)}-2\right).$$

Furthermore, $N_{i}$ orbits each transmit flows to $\frac{T_{2}}{n_{\left(1,2\right)}}$ layer-2 satellites via key satellites in Group $\frac{b_{\left(1,2\right)}}{2}$. The total flows through all marked ISLs (Figure A3) are therefore

$$\chi_{ao22sum}^{1}\left(i\right)=\frac{1}{8}T_{1}T_{2}\left(1-\frac{2}{b_{\left(1,2\right)}}\right).$$

The next step analyzes the allocation of $\chi_{ao22sum}^{1}\left(i\right)$. By symmetry, we distribute $\chi_{ao22sum}^{1}\left(i\right)$ uniformly across all N(i) intra-orbit ISLs, minimizing the maximum per-ISL load:

$$\chi_{ao22}^{1}\left(i\right)=\frac{\chi_{ao22sum}^{1}\left(i\right)}{N(i)}.$$

However, a threshold $n_{\left(1,2\right)}^{*}$ exists: for $n_{\left(1,2\right)}>n_{\left(1,2\right)}^{*}$, flows in $\chi_{ao22}^{1}\left(i\right)$ can bypass bottleneck links via ‘detour’ routes. Observing Figure A2, if the intra-orbit ISLs of the red key satellite become bottlenecked, some flows can be rerouted through alternative paths. For example, when the yellow satellite sends flows to the red satellite, some flows originally routed through the green path may be diverted through the yellow path. This ‘detour’ increases load on other link types (e.g., inter-orbit ISLs). Because $\chi_{ao22}^{1}\left(i\right)$ constitutes a small fraction of the total $\chi_{ao}^{1}\left(i\right)$, threshold $n^{*}$ is typically small for most topologies. Therefore, for simplicity, $\chi_{ao22}^{1}\left(i\right)$ is obtained by uniformly dividing $\chi_{ao22sum}^{1}\left(i\right)$ across all N(i) intra-orbit ISLs.

## Appendix D. Proof for $\chi_{aoj}^{2}\left(i\right)$ *j*∈[1, 7]

The analysis of $\chi_{ao1}^{2}$ is identical to that in Section 3.2.1. Each intra-orbit ISL carries $\frac{N_{i}M_{i}^{2}}{8}$ flows, and with $N_{i}$ such ISLs, we obtain

$$\chi_{ao1}^{2}\left(i\right)=\frac{N_{i}^{2}M_{i}^{2}}{8}.$$

For $\chi_{ao2}^{2}\left(i\right)$, a total of $\sum_{t=1}^{i-1}T_{t}$ lower-layer satellites and $\sum_{t=i+1}^{L}T_{t}$ upper-layer satellites exchange flows via $\kappa_{\left(i,i+1\right)}$. The analyses of $\chi_{ao3}^{2}\left(i\right)$, $\chi_{ao6}^{2}\left(i\right)$, and $\chi_{ao7}^{2}\left(i\right)$ parallel that of $\chi_{ao2}^{2}\left(i\right)$. The analyses of $\chi_{ao4}^{2}\left(i\right)$ and $\chi_{ao5}^{2}\left(i\right)$ follow Section 3.2.1.

## References

[1] Elbert B. (2003). The Satellite Communication Applications Handbook.

[2] Wong E. (2011). Next-generation broadband access networks and technologies. J. Light. Technol..

[3] Gandotra P., Jha R.K., Jain S. (2017). Green communication in next generation cellular networks: A survey. IEEE Access.

[4] Kodheli O., Lagunas E., Maturo N., Sharma S.K., Shankar B., Montoya J.F.M., Duncan J.C.M., Spano D., Chatzinotas S., Kisseleff S. (2021). Satellite Communications in the New Space Era: A Survey and Future Challenges. IEEE Commun. Surv. Tutor..

[5] Mortari D., Wilkins M.P. (2008). Flower constellation set theory. Part I: Compatibility and phasing. IEEE Trans. Aerosp. Electron. Syst..

[6] Ma J., Hu Z., Zhang Y., Lu Z., Wen X. Coverage Analysis Under Multi-Altitude Orbits for Multi-layer Low Earth Orbit Satellite Constellations Using Stochastic Geometry. Proceedings of the 2024 IEEE 35th International Symposium on Personal, Indoor and Mobile Radio Communications (PIMRC).

[7] Cao Q., Wang R., Ma R., Liu G., Kang W., Meng W. (2025). On Dense LEO Constellation Design for Intersatellite Interference Mitigation. IEEE Internet Things J..

[8] Ying M., Chen X., Qi Q., Zhang Z. (2026). QoS-Driven Satellite Constellation Design for LEO Satellite Internet of Things. IEEE Trans. Wirel. Commun..

[9] Gupta P., Kumar P.R. (2000). The capacity of wireless networks. IEEE Trans. Inf. Theory.

[10] Romero-Garcia J., de Gaudenzi R. (2000). On antenna design and capacity analysis for the forward link of a multibeam power controlled satellite CDMA network. IEEE J. Sel. Areas Commun..

[11] Ruan Y., Li Y., Wang C.X., Zhang R., Zhang H. Effective capacity analysis for underlay cognitive satellite-terrestrial networks. Proceedings of the 2017 IEEE International Conference on Communications (ICC).

[12] Dai C.Q., Zhang M., Li C., Zhao J., Chen Q. (2021). QoE-Aware Intelligent Satellite Constellation Design in Satellite Internet of Things. IEEE Internet Things J..

[13] Okati N., Riihonen T. (2023). Stochastic Coverage Analysis for Multi-Altitude LEO Satellite Networks. IEEE Commun. Lett..

[14] Jiang C., Zhu X. (2020). Reinforcement Learning Based Capacity Management in Multi-Layer Satellite Networks. IEEE Trans. Wirel. Commun..

[15] Zhou D., Sheng M., Wu J., Li J., Han Z. (2022). Gateway Placement in Integrated Satellite-Terrestrial Networks: Supporting Communications and Internet of Remote Things. IEEE Internet Things J..

[16] Dai C.Q., Yu T., Chen Q. Capacity-Oriented Satellite Constellation Design in Disaster Emergency Communication Network. Proceedings of the 2020 International Conference on Wireless Communications and Signal Processing (WCSP).

[17] Zhou D., Sheng M., Wang X., Xu C., Liu R., Li J. (2017). Mission Aware Contact Plan Design in Resource-Limited Small Satellite Networks. IEEE Trans. Commun..

[18] Sun J., Modiano E. (2003). Capacity provisioning and failure recovery for Low Earth Orbit satellite constellation. Int. J. Satell. Commun. Netw..

[19] Guo L., Liu J., Sheng M., Li J. (2025). Constellation Topology Design for Maximum Capacity of LEO Satellite Networks. IEEE Trans. Commun..

[20] Lan T., Zhou D., Sheng M., Li J. (2025). Capacity Analysis of LEO Mega-Constellations with Quasi-Torus Topologies. IEEE Trans. Commun..

[21] Song Z., An J., Pan G., Wang S., Zhang H., Chen Y., Alouini M.S. (2023). Cooperative Satellite-Aerial-Terrestrial Systems: A Stochastic Geometry Model. IEEE Trans. Wirel. Commun..

[22] Liu R., Sheng M., Lui K.S., Wang X., Zhou D., Wang Y. Capacity Analysis of Two-Layered LEO/MEO Satellite Networks. Proceedings of the 2015 IEEE 81st Vehicular Technology Conference (VTC Spring).

[23] Hemmati H. (2006). Deep Space Optical Communications.

[24] Zhu X., Jiang C., Kuang L., Dong M., Zhao Z. Capacity Analysis of Multi-layer Satellite Networks. Proceedings of the 2020 International Wireless Communications and Mobile Computing (IWCMC).

[25] Liu R., Wu W., Yang Q., Zhou D., Zhang W. (2020). Exploring the Information Capacity of Remote Sensing Satellite Networks. IEEE Access.

[26] Lei L., Wang A., Lagunas E., Hu X., Zhang Z., Wei Z., Chatzinotas S. (2024). Spatial–Temporal Resource Optimization for Uneven-Traffic LEO Satellite Systems: Beam Pattern Selection and User Scheduling. IEEE J. Sel. Areas Commun..

[27] Chen Q., Yang L., Zhao Y., Wang Y., Zhou H., Chen X. (2024). Shortest Path in LEO Satellite Constellation Networks: An Explicit Analytic Approach. IEEE J. Sel. Areas Commun..

[28] Chen Q., Giambene G., Yang L., Fan C., Chen X. (2021). Analysis of Inter-Satellite Link Paths for LEO Mega-Constellation Networks. IEEE Trans. Veh. Technol..

[29] Gao Y., Chiu D.M., Lui J.C. Determining the end-to-end throughput capacity in multi-hop networks: Methodology and applications. Proceedings of the Joint International Conference on Measurement and Modeling of Computer Systems.

